# Open Sesame: treasure in store-operated calcium entry pathway for cancer therapy

**DOI:** 10.1007/s11427-014-4774-3

**Published:** 2014-12-07

**Authors:** PAN Zui, MA JianJie

**Affiliations:** 1 Department of Internal Medicine, The Ohio State University Wexner Medical Center, Columbus, OH 43210, USA; 2 Department of Surgery, The Ohio State University Wexner Medical Center, Columbus, OH 43210, USA; 3 Comprehensive Cancer Center, The Ohio State University Wexner Medical Center, Columbus, OH 43210, USA; 4 Davis Heart and Lung Research Institute, The Ohio State University Wexner Medical Center, Columbus, OH 43210, USA

**Keywords:** Orai1, STIM1, Ca^2+^ oscillations, proliferation, metastasis, oncogenic

## Abstract

Store-operated Ca^2+^ entry (SOCE) controls intracellular Ca^2+^ homeostasis and regulates a wide range of cellular events including proliferation, migration and invasion. The discovery of STIM proteins as Ca^2+^ sensors and Orai proteins as Ca^2+^ channel pore forming units provided molecular tools to understand the physiological function of SOCE. Many studies have revealed the pathophysiological roles of Orai and STIM in tumor cells. This review focuses on recent advances in SOCE and its contribution to tumorigenesis. Altered Orai and/or STIM functions may serve as biomarkers for cancer prognosis, and targeting the SOCE pathway may provide a novel means for cancer treatment.

The discovery of STIM1 and Orai1 genes reminds us the old folk tale *Ali Baba and Forty Thieves* in *One Thousand and One Nights*. In that story, when Ali Baba spits out the magical phrase “Open Sesame”, it unlocks the gate of a cave where the thieves hide their treasures. Would Orai/STIM-mediated SOCE lead us to the treasure for new diagnostic and prognostic tools, or even novel means for cancer treatment?

## 1 Ca^2+^ signaling and store-operated Ca^2+^ entry machinery

Ca^2+^, the mighty signaler, regulates a wide range of downstream cellular processes, including gene transcription, cell proliferation, migration and death [1–6]. It has long been recognized that dysregulation of Ca^2+^ homeostasis is associated with a plethora of pathological conditions including immune deficiency, neurodegeneration, muscular and cardiovascular disorders as well as cancer progression. The altered Ca^2+^ signaling may contribute to tumor angiogenesis, progression, and metastasis. Searching for specific genes that contribute to altered Ca^2+^ signaling in tumor cells has emerged as an exciting area in cancer research [7–15].

**Figure 1:**
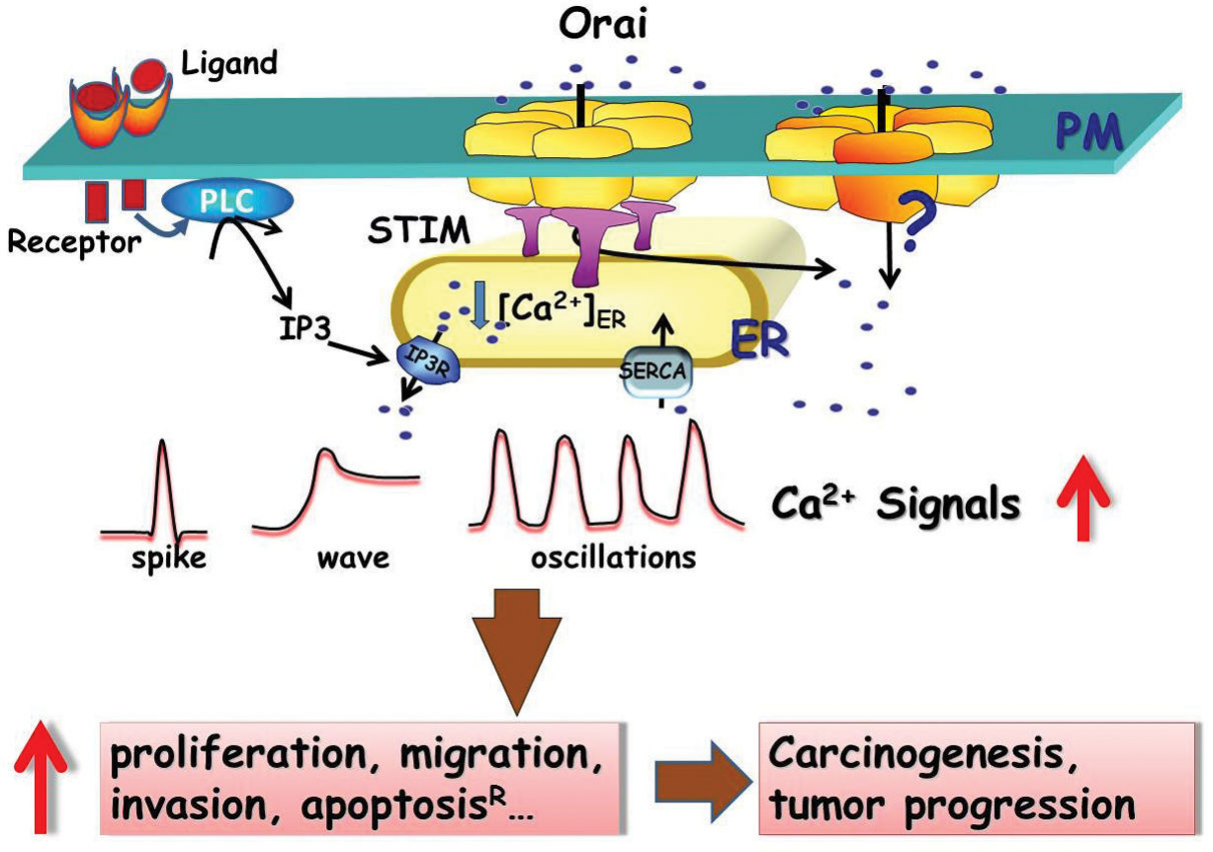
Oncogenic function of Orai1 and STIM1. Intracellular Ca^2+^ signals are in the forms of spike, wave or oscillation, which require G-protein coupled receptor-PLC-IP_3_R pathway and Orai1/STIM1-mediated SOCE. Orai complex is composed of six subunits, coupling with STIM1 with 2:1 ratio for optimal channel regulation and activation. In cancer cells, elevated expression of Orai1 (Orai3) is not always accompanied by upregulation of STIM1, which raises the question whether Orai1 (Orai3) channel is activated without coupling with STIM1. Nevertheless, the overall increased SOCE result in hyperactivity of intracellular Ca^2+^ signals, which in turn stimulate cell proliferation, migration/invasion and develop apoptotic resistance in cancer cells.

Intracellular Ca^2+^ signaling is a complex and fine-tuning network. The spatially-temporally confined Ca^2+^ signaling is tightly regulated in the form of waves, spikes or oscillations (Figure 1). Intracellular Ca^2+^ oscillation is a remarkable process, since its frequency, amplitude and duration can act as “calcium code*”* to activate transcription factors for gene transcription, cell proliferation and migration [16–18]. Ca^2+^ signaling is orchestrated with the release of Ca^2+^ through IP_3_ receptor from internal Ca^2+^ stores such as endoplasmic reticulum (ER), the uptake of Ca^2+^ into the ER, and Ca^2+^ influx across plasma membrane (PM) from extracellular Ca^2+^ reservoir. The latter is mainly mediated by a process named store-operated Ca^2+^ entry (SOCE) [19–24]. SOCE was first described by Jim Putney about three decades ago who coined the term capacitative Ca^2+^ entry (CCE) [20]. In this pathway, activation of the G-protein coupled receptor leads to stimulation of PLC to generate IP_3_; IP_3_ in turn causes intracellular Ca^2+^ release that is followed by reduction of Ca^2+^ concentration inside the ER lumen. The reduced ER Ca^2+^ store sends a signal to the PM to activate CCE, allowing refill of the empty ER Ca^2+^ stores (Figure 1). Using the whole-cell patch clamp technique, Hoth et al. [25] characterized the electrophysiological property of Ca^2+^ release-activated Ca^2+^ current (CRAC), which mediates SOCE in mast cells. The molecular players mediating SOCE were discovered rather recently. Using RNA interference (RNAi)-based screening, two independent groups identified stromal interaction molecule 1 (STIM1) as the ER Ca^2+^ sensor [26–28]. Shortly after, a genome-wide screening using the severe combined immune deficiency (SCID) disease model that is caused by defective Ca^2+^ entry in T cells led to a groundbreaking discovery of a membrane protein as the SOCE channel [29–32]. This protein is named Orai, a mythological character that served as gate keeper to safeguard the path toward heaven. STIM1 is an ER-resident membrane protein, containing a luminal EF-hand allowing it to detect changes in the ER Ca^2+^ content. Orai1 is an integral membrane protein with four trans-membrane domains that constitutes the pore forming unit of the SOCE channel. Upon ER Ca^2+^ store depletion or reduction in some cases, STIM1 molecules cluster at the ER/PM junctional region [21,26,28,29,33–39], where they send retrograde signals to Orai1 for opening of the Ca^2+^ channels [33,40,41]. In addition to STIM1 and Orai1, mammalian genomes also encode an additional STIM homologue, STIM2; and two Orai1 homologues, Orai2 and Orai3. Extensive studies from CRAC channels in immune system support that Orai1 and STIM1 are necessary and sufficient for the assembly and activation of the “classical” SOCE complex.

## 2 Emerging role of SOCE in cancer biology

Long before the discovery of *Orai* and *Stim* genes, pharmacological studies have revealed the important role of SOCE in cancer cells. Carboxyamidotriazole is an anti-cancer drug [42] that inhibits angiogenesis due to its ability to target SOCE in many carcinoma cell lines [43–47]. Other SOCE blockers, such as 2-aminoethyl diphenylborate (2-APB) and SKF-96365, were reported to have similar effects on cancer cells. For example, 2-APB inhibits proliferation in human hepatoma HepG2 and Huh-7 cells, lung cancer A549 cells and colon cancer T84 cells [47–50]. Using these pharmacological tools, several studies showed that altered function of SOCE might be a general phenomenon associated with cancer progression [51–54]. Our earlier studies showed a functional interaction between pro- apoptotic protein Bax and SOCE in apoptosis of prostate cancer, suggesting that the androgen-independent prostate epithelial tumor cells could gain their apoptotic resistance by down-regulation of SOCE [55,56]. SOCE may also cross-talk to signaling cascades initiated by tumorigenetic and angiogenic growth factors. For example, epidermal growth factor (EGF) is known to stimulate intracellular Ca^2+^ release through PLC/IP_3_ pathway leading to activation of SOCE (as illustrated in Figure 1). It was proposed that non-steroidal anti-inflammatory drugs inhibit colorectal carcinogenesis by attenuation of EGF-induced cellular proliferation through a process independent of their inhibitory effect on prostaglandin synthesis but rather by blocking EGF-induced SOCE [57].

## 3 Different functions of STIM and Orai proteins in different cancers

The discovery of STIM1 and Orai1 as the molecular components of SOCE has greatly advanced our understanding of the pathophysiological roles of SOCE in cancer. While genetic mutations in Orai1 or STIM1 were linked to immune disorders, skeletal muscle myopathy and heart hypertrophy [7,58–63], the functions of these genes in various types of cancer have fascinated many investigators. Combination of pharmacologic and siRNA-mediated gene knockdown approaches has pinpointed the involvement of Orai1 and STIM1 in five key events of tumorigenesis: elevated proliferation, enhanced migration/invasion, increased resistance to apoptosis, angiogenic switch and reduction in antitumor immunity. Orai1 and STIM1 were reported to promote cell proliferation, migration, invasion and apoptotic resistance in breast cancer [15], glioblastoma [64], prostate cancer [65–67], hepatocellular carcinoma [14], esophageal squamous cell carcinoma (ESCC) [68] and clear cell renal cell carcinoma (ccRCC) [69]. They are also required for the anti-tumor activity of cytotoxic T cells, i.e., secretion of cytokines, such as TNFα, IL-2 and IFNg, which induce apoptosis of cancer cells [70,71].

We recently undertook a study to investigate the clinical significance of Orai1 and STIM1 in esophageal cancer. That study showed that expression of Orai1 in tumors obtained from patients with ESCC was significantly elevated compared with that in neighboring non-tumorous esophageal tissues [68]. High Orai1 expression was associated with the recurrence rate for this disease independent of other variables. This study provided the first evidence in support of an association between Orai1 expression and the clinical outcome of cancer patients. At about the same time as the publication of our study, a Korean group reported a similar observation that Orai1 is overexpressed in tumor tissues from patients with ccRCC [69]. Thus, these results raise the possibility that Orai1 expression could be a potential prognostic biomarker for ESCC or ccRCC. It should be mentioned that STIM1 was reported to be overexpressed in tumor tissues from subjects with early-stage cervical cancer [9]. However, the study from our group and the Korean group showed that the expression of STIM1 in tumor tissues remained unchanged or was even reduced as compared to that in neighboring normal tissues based on real-time RT-PCR, Western blot and immunohistochemistry assays. These findings suggest that variations of SOCE components and regulatory mechanisms may be different in different types of cancers. Feng et al. [11] identified a signaling pathway in which formation of an Orai1-SPCA2 complex elicits a constitutive store-independent Ca^2+^ entry pathway that regulates tumor-igenesis in breast cancer. Such Ca^2+^ entry pathway appears to be mediated by Orai3 in estrogen receptor-positive breast cancer and non-small cell lung carcinoma cells, whereas the “classical” STIM1/Orai1 pathway predominates in estrogen receptor-negative breast cancer cells [72,73]. In addition, Chantome et al. [74] showed that knockdown of STIM1 had no effect on, whereas knockdown of Orai1 inhibited, migration of breast cancer cells, indicating STIM1 might not be involved in the metastatic process. Our observation of significantly higher expression of STIM2 in ESCC cells implies that STIM2 may play a role in regulation of Orai channel activity and overall intracellular Ca^2+^ signaling in this malignancy [68].

Functional ion channels often form from subunits assembled into homo- or hetero-multimers. Different stoichiometry of Orai1 and STIM1 may result in different channel property and regulatory mechanism. The crystal structure of Orai protein revealed a hexametric structure for the functional channel through coupling with STIM1 [75], and provided evidence to support the model that the optimal Orai1/STIM1 for maximal SOCE activation is 2:1 [76]. Variations in the ratios of Orai1 to STIM1 were reported in different cell types [77]. Dubois et al. [67] showed that varying Orai1/Orai3 ratios modulate the function of SOCE and basal cytosolic Ca^2+^ level, and Orai3 overexpression stimulates cell proliferation and promotes apoptosis resistance in prostate cancer cells. They proposed that remodeling of Orai1/Orai3 may constitute as an oncogenic switch in prostate cancer. Wang et al. [78] identified distinct Orai1- coupling domains in STIM1 and STIM2, which determines the efficacy of interaction between Orai1 and STIM as well as its store-dependent activation properties. In our study, we found an increased expression of Orai1 and STIM2 but not STIM1 in ESCC tumor tissues [68]. Thus, it is possible that STIM2 may replace STIM1 to couple with Orai1 in ESCC cells. Clearly, further studies are required to define the mechanisms underlying the role of specific STIMs and Orais in regulating the overall function of SOCE. Targeting these specific properties may be used for prevention and/or treatment of cancers.

## 4 Targeting SOCE-mediated Ca^2+^ signaling in cancer therapy

SOCE machinery as a signaling complex contains several components, e.g., Orai1, Orai3, STIM1 and STIM2. Altered expression of these signaling components may underlie the altered function of SOCE, which in turn activates the downstream cellular events for carcinogenesis and tumor progression (Figure 1). Compared to quiescent non-tumor cells, we identified a striking hyperactivity in Ca^2+^ oscillations in ESCC cells, which is dependent on Orai1-mediated SOCE since these oscillations could be suppressed by reduction of Orai1 function using either pharmacologic or molecular approaches [68]. In the same study, we also showed that inhibition of Orai1-mediated SOCE by pharmacologic antagonists of the channel or reduction of Orai1 expression by Orai1 knockdown impeded the proliferation and migration/invasion of ESCC cells *in vitro*, suppressed the tumor growth *in vivo*. Similar molecular and pharmacologic approaches employed have been utilized by other investigators to evaluate the importance of STIM1, Orai1 or Orai3 to the migration and metastasis of breast, cervical, prostate and renal carcinoma [9,15,67,69,72,73]. All these studies compiled proof of principle for targeting SOCE channel in cancer treatments. For example, injection of SKF-96365 into xenografted breast, cervical and esophageal cancer mice models suppressed tumor growth, angiogenesis and metastasis *in vivo* [9,15,68]. Clearly, a better understanding of the regulatory mechanism underlying Orai-mediated SOCE in different cancers, and the development of specific/potent SOCE channel modulators will greatly advance this field.

## 5 Summary

Over the past few years we have gained significantly the basic molecular insight of Orai/STIM-mediated SOCE. However, many questions regarding the contribution of STIM and Orai proteins to carcinogenesis and tumor progression remain. Understanding of the mechanisms by which STIM and Orai proteins exert their different patho-physiological roles in different cancers will be a major challenge over the coming years. Orai channels constitute potential therapeutic targets for treatment of human diseases. For millions of cancer patients, we have high expectation that Orai-mediated SOCE pathway may represent the magical code to unlock the gate toward new diagnostic and prognostic means or treatment strategies for combating cancers.
